# Gut Microbiota-Derived Metabolites in Atherosclerosis: Pathways, Biomarkers, and Targets

**DOI:** 10.3390/ijms26178488

**Published:** 2025-09-01

**Authors:** Alexandra-Kristine Tonch-Cerbu, Adrian-Gheorghe Boicean, Oana-Maria Stoia, Minodora Teodoru

**Affiliations:** 1 Medical Clinical Department, Faculty of Medicine, “Lucian Blaga” University, 550024 Sibiu, Romania; 2 County Clinical Emergency Hospital of Sibiu, 550245 Sibiu, Romania

**Keywords:** gut microbiota, metabolites, atherosclerosis, dysbiosis, biomarkers, probiotics, prebiotics, therapeutic strategies

## Abstract

The human gut microbiota is a complex ecosystem that influences host metabolism, immune function, and cardiovascular health. Dysbiosis, defined as an imbalance in microbial composition or function, has been linked to the development and progression of atherosclerosis. This connection is mediated by microbial metabolites that enter the systemic circulation and interact with vascular and immune pathways. Among these, trimethylamine N-oxide (TMAO) has been most extensively studied and is consistently associated with cardiovascular events. Other metabolites, including lipopolysaccharides (LPS), short-chain fatty acids (SCFAs), and secondary bile acids, also contribute by modulating inflammation, endothelial function, and lipid metabolism. Recent research has expanded to emerging metabolites such as indoxyl sulfate, indole-3-propionic acid, and polyamines, which may provide additional mechanistic insights. These microbial products are increasingly explored as biomarkers of cardiovascular risk. TMAO has shown predictive value in large human cohorts, while microbiota composition and diversity measures remain less consistent across studies. However, interpretation of these biomarkers is limited by methodological variability, interindividual differences, and lack of standardization. Therapeutic interventions targeting the gut–heart axis are under investigation. Dietary strategies such as the Mediterranean diet and fiber-rich nutrition, probiotics and prebiotics, and fecal microbiota transplantation (FMT) show promise, while pharmacological approaches targeting TMAO or bile acid pathways are in early stages. This review summarizes current knowledge on the mechanistic, diagnostic, and therapeutic links between the gut microbiota and atherosclerosis, highlighting both established findings and emerging directions for future research.

## 1. Introduction

The understanding of atherosclerosis has evolved significantly since the early 19th century, beginning with Karl von Rokitansky’s observation of arterial wall thickening due to intraluminal deposits. Ludwig Aschoff later introduced the first classification of atherosclerotic plaques, identifying their lipidic and fibrotic nature. In the 1950s, the WHO defined “complicated plaques” with features such as thrombosis and ulceration. The American Heart Association refined classification in 1994 into eight lesion types, and in the early 2000s, Virmani et al. proposed a clinically oriented model with four main lesion categories, highlighting the dynamic progression of atherosclerotic disease.

Atherosclerosis is now widely recognized as a chronic inflammatory disease of the arterial wall, contributing to approximately 50% of all deaths in industrialized societies. It is fundamentally a lipid-driven process, initiated by the subendothelial accumulation of low-density lipoproteins (LDL) and remnant lipoprotein particles, coupled with localized vascular inflammation. This pathophysiological sequence underlies the development of atherosclerotic cardiovascular disease (ASCVD), which encompasses myocardial infarction, ischemic stroke, and peripheral arterial disease [1,2,3].

Cardiovascular disease, primarily driven by atherosclerosis, has been the subject of extensive investigation, yielding critical insights into cholesterol biosynthesis, lipoprotein metabolism, and associated risk factors. Atherosclerosis, the predominant cause of ischemic heart disease, affects large and medium-sized arteries, with lesion development favoring regions exposed to low or oscillatory shear stress. It is a multifactorial pathology shaped by both environmental influences and genetic predispositions.

Atherosclerotic lesion progression is characterized by a transition from early lipid-rich fatty streaks to structurally complex fibrous plaques, which may ultimately lead to luminal narrowing, vascular occlusion, and acute cardiovascular events. These lesions arise within the intimal layer of the arterial wall which, together with the media and adventitia, comprises the three-layered architecture of susceptible vessels. Atherogenesis is initiated by endothelial dysfunction, which increases vascular permeability and facilitates the subendothelial accumulation of lipids and immune cells.

Lipoproteins play a pivotal role in this process, particularly low-density lipoproteins (LDL). Elevated plasma levels of LDL, and more critically, its oxidized form (oxLDL), promote vascular inflammation and plaque formation. Oxidative stress marked by an imbalance between pro-oxidant species, such as reactive oxygen species (ROS) and endogenous antioxidant systems, further exacerbates endothelial injury and lipid peroxidation. ROS not only directly damage vascular cells but also modulate vasoactive molecules and inflammatory pathways.

The immunoinflammatory component of atherosclerosis involves recruitment and activation of monocytes and T lymphocytes, which migrate into the intima and differentiate into foam cells upon uptake of modified lipoproteins. Foam cells, in turn, secrete pro-inflammatory cytokines and matrix-degrading enzymes, contributing to plaque expansion and instability. Adhesion molecules and chemokines amplify leukocyte recruitment, sustaining a chronic inflammatory milieu.

Endothelial cells serve as mechanosensitive regulators of vascular homeostasis and are profoundly influenced by hemodynamic forces. While laminar shear stress exerts a protective effect by maintaining an anti-inflammatory and antithrombotic endothelial phenotype, disturbed flow patterns promote the activation of pro-atherogenic transcription factors, such as NF-κB, which orchestrate inflammatory gene expression and endothelial dysfunction.

Foam cell formation remains a hallmark of atherosclerotic pathology, representing the intersection of lipid metabolism and innate immune activation. The accumulation of foam cells and their secretory products alters the composition and stability of the arterial wall, accelerating disease progression.

**Figure 1:**
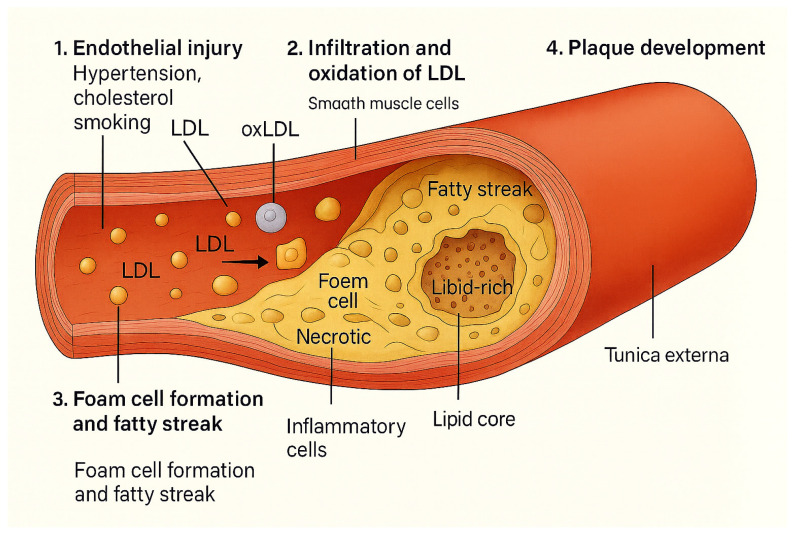
Classical pathogenesis of atherosclerosis. Endothelial injury allows LDL infiltration and oxidation, leading to macrophage uptake, foam cell formation, fatty streaks, and eventually plaque development with inflammatory and necrotic components.

Figure 1 illustrates the classical stages of atherosclerosis, beginning with endothelial injury and LDL infiltration, followed by oxidation and foam cell formation, and culminating in plaque development. This provides a general framework onto which the modulatory effects of gut microbiota-derived metabolites (TMAO, LPS, SCFAs, bile acids) can be mapped in subsequent sections.

A comprehensive understanding of the cellular and molecular mechanisms underlying atherosclerosis is essential for the identification of novel therapeutic targets and the development of effective strategies for the prevention and management of cardiovascular disease [4,5].

Atherosclerosis is a highly modifiable condition, with its progression strongly influenced by dietary patterns and lifestyle behaviors. Interventions such as the adoption of a structured lifestyle, regular physical activity, smoking cessation, and adherence to a nutrient-rich diet are fundamental strategies for symptom management and the enhancement of cardiovascular health and quality of life. Among these, diet plays a pivotal role, as nutritional intake significantly impacts lipid metabolism, systemic inflammation, and plaque stability [6].

Excessive intake of saturated fats and dietary cholesterol is associated with elevated plasma cholesterol levels, contributing to atherogenesis. Conversely, the consumption of polyunsaturated fatty acids (PUFAs), particularly n-3 (omega-3) and n-6 (omega-6) fatty acids, has demonstrated cholesterol-lowering effects and is associated with a reduced risk of atherosclerosis. In contrast, trans fatty acids are strongly correlated with adverse cardiovascular outcomes and should be minimized in dietary practices [7].

Cholesterol homeostasis, regulated by a complex interplay of endogenous biosynthesis and dietary absorption, is modifiable through both nutritional strategies and pharmacological interventions. Functional dietary components such as PUFAs, phytosterols, polyphenols, and essential vitamins have garnered increasing attention for their cardioprotective properties. These compounds exert anti-inflammatory, antioxidant, and lipid-lowering effects, thereby contributing to atherosclerosis prevention and plaque stabilization [8,9].

The gut microbiota comprises a diverse community of microorganisms including bacteria, viruses, fungi, and archaea, with bacteria being the most abundant. These microbes primarily inhabit the gastrointestinal tract, especially the nutrient-rich, anaerobic environment of the ascending colon [10].

In infants, gut microbiota composition is influenced by the mode of delivery. Vaginally delivered infants are initially colonized by *Lactobacillus* and *Prevotella*, while those born via cesarean section harbor skin-associated bacteria such as *Streptococcus*, *Corynebacterium*, and *Propionibacterium*, making them potentially more susceptible to infections. For instance, 64–82% of newborns with MRSA infections were delivered by C-section. The gut microbiota evolves during early life, reaching adult-like complexity and stability by the age of three. Diet (breast milk vs. formula) and antibiotic exposure further shape microbial development, with long-term health implications [11].

In adults, the dominant gut microbiota phyla include Firmicutes, Bacteroidetes, Actinobacteria, Proteobacteria, and Verrucomicrobia, with Firmicutes and Bacteroidetes comprising over 90% of the community. The Firmicutes/Bacteroidetes (F/B) ratio is often used as a health marker; it is elevated in obesity and cardiovascular disease. Studies have shown an increased Firmicutes and decreased Bacteroidetes abundance in patients with coronary artery disease and hypertension.

Gut microbiota composition also shifts with aging, marked by reduced microbial diversity and a decline in beneficial species. These changes may contribute to the development of age-related diseases such as atherosclerosis [12].

The global rise in immune-mediated, metabolic, and neurological diseases is increasingly linked to gut microbiota dysbiosis, an imbalance in microbial composition marked by reduced diversity, loss of beneficial microbes, and overgrowth of harmful ones. Environmental factors such as diet, medications, and food additives play a significant role in disrupting the gut microbiota, leading to impaired gut barrier integrity, immune dysregulation, and metabolic disturbances [13].

The term dysbiosis, first introduced by Scheunert in 1920 and later refined by Haenel to contrast with the healthy state of eubiosis, is commonly used to describe an imbalance in gut microbial composition [14]. In contemporary microbiome research, dysbiosis generally refers to alterations in the diversity, abundance, or function of microbial communities that are associated with disease. However, its definition remains inconsistent, being applied to general shifts, loss of homeostasis, or specific taxonomic changes [15].

Dysbiosis is associated with numerous diseases, including obesity, diabetes, IBD, cardiovascular disease, liver disorders, and COVID-19. Pathogenic changes in microbiota composition often precede disease onset, suggesting a possible causative role. Studies have identified microbial signatures and metabolites (SCFAs, TMA, succinate) as potential diagnostic and prognostic biomarkers [16].

Therapeutic strategies to restore microbiota balance include probiotics, prebiotics, fecal microbiota transplantation (FMT), and targeted metabolic modulation. FMT is highly effective in treating recurrent *Clostridioides difficile* infections and is being explored for other conditions. Probiotics like *Lactobacillus* and *Faecalibacterium* show promise in managing inflammation and improving gut health, but personalized approaches and high-quality clinical trials are needed [17].

Targeting microbial metabolites such as inhibiting TMA production to reduce cardiovascular risk or enhancing SCFA levels to improve liver health represents a growing therapeutic frontier. Further research is needed to clarify whether dysbiosis is a cause or consequence of disease, but mounting evidence supports its central role in pathogenesis and treatment across multiple disease domains [18].

Atherosclerosis is a chronic inflammatory condition strongly linked to gut dysbiosis and increased intestinal permeability. The gut epithelium acts as a critical barrier that prevents the translocation of microbial components into circulation. Disruption of this barrier, marked by decreased expression of tight junction proteins, allows pathogen-associated molecular patterns (PAMPs), such as lipopolysaccharide (LPS) and peptidoglycan (PG), to enter the bloodstream and trigger systemic inflammation [19].

LPS, a component of Gram-negative bacteria, is closely associated with cardiovascular disease (CVD). It activates immune signaling via Toll-like receptor 4 (TLR4) and its co-receptors, promoting inflammatory pathways (NF-κB, MyD88) and increasing cytokine production (IL-6, IL-1, TNF-α). Elevated LPS levels due to gut dysbiosis have been detected in individuals with higher CVD burden. While some genetic polymorphisms in TLR4 show inconclusive effects on atherosclerosis, TLR4 or MyD88 deficiency in animal models reduces plaque formation [20].

Similarly, peptidoglycan, present in both Gram-negative and Gram-positive bacteria, activates immune responses via NOD1 and NOD2 receptors, contributing to inflammation and gut barrier dysfunction. NOD1/2 deficiency in mice reduces atherosclerosis, emphasizing the role of innate immunity in disease progression. Other microbial PAMPs (flagellin, CpG DNA, lipopeptides) also contribute to vascular inflammation through host pattern recognition receptors [21].

Collectively, these findings support the view that gut microbiota imbalance contributes to atherosclerosis through microbial translocation and immune activation. However, despite clear links between pathogenic bacteria and CVD, antibiotic trials for atherosclerosis have shown mixed results, highlighting the complexity of host–microbe interactions in cardiovascular disease.

Beyond inflammation, gut microbiota-derived metabolites play a significant role in the development of cardiovascular disease (CVD). These metabolites, including short-chain fatty acids (SCFAs), methylamines, polyamines, trimethylamine N-oxide (TMAO), and secondary bile acids (BAs), result from microbial metabolism and co-metabolism with the host. SCFAs are well known for their roles in metabolic regulation, but growing evidence links TMAO and secondary BAs specifically to the pathogenesis of atherosclerosis [22].

The objective of this review is to investigate the mechanisms by which the gut microbiota contributes to the development and progression of atherosclerosis. Emphasis is placed on the impact of gut dysbiosis in compromising intestinal barrier integrity, facilitating systemic inflammation via microbial components such as lipopolysaccharide (LPS) and peptidoglycan (PG), and activating immune signaling pathways. Additionally, the role of microbiota-derived metabolites, particularly trimethylamine N-oxide (TMAO) and secondary bile acids, in promoting atherogenesis is examined. By integrating historical perspectives on atherosclerotic plaque classification with recent advances in microbiota research, this review highlights the gut microbiota as a potential source of diagnostic markers and a promising target for therapeutic intervention in cardiovascular disease.

To provide a coherent framework, this review is structured in three parts: mechanistic insights into how microbial metabolites influence atherosclerosis, evidence for their role as biomarkers and clinical correlates, and therapeutic strategies aimed at modulating the microbiota–atherosclerosis axis.

**Figure 2:**
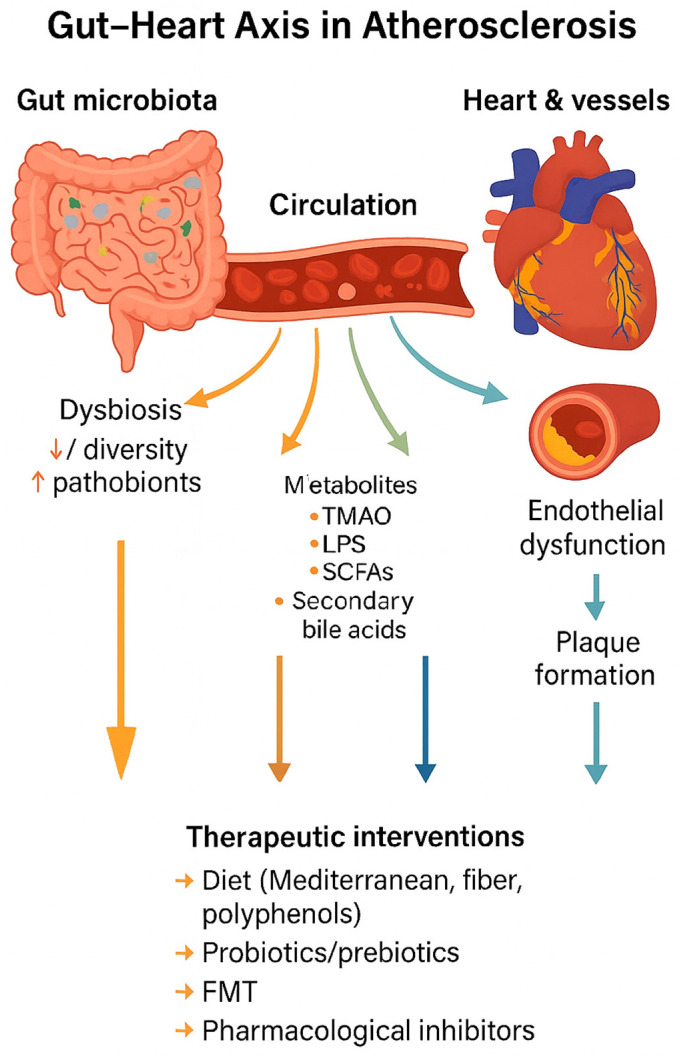
Gut–heart axis in atherosclerosis. Gut dysbiosis alters microbial metabolite production (TMAO, LPS, SCFAs, secondary bile acids), which enter circulation and drive endothelial dysfunction, inflammation, and plaque formation. Therapeutic strategies such as diet, probiotics, prebiotics, FMT, and pharmacological inhibitors can modulate this axis.

Figure 2 illustrates the conceptual framework of the gut–heart axis in atherosclerosis, summarizing how dysbiosis leads to metabolite production, systemic circulation, vascular dysfunction, and plaque formation, as well as potential therapeutic interventions.

## 2. Background

A comprehensive literature search was conducted across PubMed, Scopus, and Web of Science using terms such as “gut microbiota”, “atherosclerosis”, “metabolites”, and “therapeutic targets” to identify studies with experimental or clinical data on the gut microbiota’s involvement in atherosclerosis development and progression. Articles were selected based on topical relevance, recency, and methodological rigor, excluding those lacking direct evidence of gut microbiota involvement or with incomplete methodologies. Data were extracted regarding experimental models, effects on vascular inflammation, lipid metabolism, and therapeutic interventions. Physio-pathological effects were compared by analyzing animal and human studies.

## 3. Main Text

### 3.1. Pathogenic Mechanisms

Gut dysbiosis, defined as a disruption in the composition and function of the gut microbiota, promotes systemic inflammation a key driver of atherosclerosis through multiple interconnected mechanisms. Characterized by reduced microbial diversity, loss of beneficial species (*Akkermansia*, *Faecalibacterium*), and overgrowth of pathogenic bacteria (*Proteobacteria*), dysbiosis compromises intestinal barrier integrity by downregulating tight junction proteins, resulting in increased gut permeability. This allows the translocation of microbial components such as lipopolysaccharides (LPS) and peptidoglycans (PGs) into the systemic circulation, where they activate innate immune receptors like TLRs and NODs, triggering NF-κB signaling and the release of pro-inflammatory cytokines (IL-6, TNF-α, IL-1β).

Concurrently, gut-derived metabolites such as trimethylamine N-oxide (TMAO) and secondary bile acids (DCA, LCA) further amplify inflammation by promoting oxidative stress, adhesion molecule expression, monocyte recruitment, and endothelial dysfunction. TMAO also contributes to foam cell formation, platelet activation, and vascular permeability.

In contrast, short-chain fatty acids (SCFAs), particularly butyrate, which normally exert anti-inflammatory and barrier-protective effects, are reduced in dysbiosis, diminishing their role in regulating immune tolerance, oxidative stress, and vascular health. The cumulative effect is a chronic low-grade systemic inflammation that fuels atherogenesis by enhancing immune cell infiltration, endothelial injury, and plaque instability.

Emerging data sharpen the understanding of the microbiota–immune axis in atherosclerosis. In diet-induced models, an obesogenic, low-fiber diet reshapes the gut microbiota and promotes gut lymphocyte trafficking from mesenteric lymph nodes to the periphery, thereby exacerbating atherogenesis; interruption of this trafficking abrogates the effect [23].

Independently, intestinal tryptophan metabolism modulates immune tone and plaque formation: shifting tryptophan away from microbial indoles toward host kynurenine pathways alters mucosal systemic immunity and reduces/augments atherosclerosis, depending on the enzymatic node targeted [24].

At the population level, Mendelian randomization suggests a causal pathway in which gut microbiota influence coronary heart disease risk via immune cell mediators, supporting a mechanistic link beyond confounding [25].

Comprehensive 2024 syntheses further consolidate these connections, while also noting context dependence; for example, in intermittent hyperlipidemia, microbiota contributions to plaque acceleration may be limited [26,27].

Thus, targeting gut microbiota composition and function through dietary modulation, probiotics, or inhibition of pathogenic microbial metabolites may represent a promising therapeutic strategy in reducing vascular inflammation and preventing atherosclerosis [28,29].

#### 3.1.1. Lipopolysaccharides (LPS)

Lipopolysaccharides (LPS), major components of Gram-negative bacterial walls, are potent inducers of systemic inflammation. Their pro-atherogenic action is mainly mediated by binding to TLR4 on endothelial cells and macrophages, activating NF-κB and the NOD-, LRR- and pyrin domain-containing protein 3 (NLRP3) inflammasome, which in turn stimulate cytokine release (IL-6, TNF-α, IL-1β). These effects promote endothelial dysfunction, monocyte recruitment, and plaque progression [30,31,32,33,34,35,36,37,38,39].

LPS, a component of Gram-negative bacterial membranes, induces systemic inflammation by binding to TLR4 on endothelial cells and macrophages, triggering NF-κB activation and cytokine release [40]. Clinically, endotoxemia has been associated with carotid atherosclerosis and cardiovascular events in prospective population studies, such as the Bruneck cohort [41]. These findings support a mechanistic and epidemiological link between low-grade endotoxemia and atherosclerosis.

While consistent in experimental models, the clinical impact of circulating LPS remains debated, as low-grade endotoxemia is often confounded by metabolic and renal disorders. Thus, LPS represent a mechanistically plausible but clinically variable contributor to atherosclerosis [42,43].

#### 3.1.2. Trimethylamine N-Oxide (TMAO)

Trimethylamine-N-oxide (TMAO), generated from dietary choline and L-carnitine by gut microbiota and hepatic FMO3, is one of the most extensively studied microbial metabolites in cardiovascular disease. Mechanistically, TMAO enhances foam cell formation by upregulating scavenger receptors (CD36, SR-A1) and impairing cholesterol efflux (ABCA1, ABCG1), promotes platelet hyperreactivity through MAPK and Ca^2+^ signaling, and augments vascular inflammation [29].

TMAO contributes to atherosclerosis through multiple mechanisms. It promotes foam cell formation by upregulating the macrophage scavenger receptors CD36 and SR-A1, facilitating oxLDL uptake [29]. TMAO also enhances platelet hyperreactivity and thrombosis risk via calcium signaling and MAPK activation [44]. Additionally, it augments endothelial inflammation by activating NF-κB and altering bile acid metabolism [45]. These mechanistic insights provide a strong biological rationale for the observed association between elevated TMAO levels and adverse cardiovascular outcomes. These mechanistic findings have been validated and expanded in subsequent studies, linking TMAO to vascular inflammation and adverse cardiovascular outcomes [46,47,48,49].

Although multiple clinical studies link elevated TMAO to increased cardiovascular risk, recent large cohorts suggest heterogeneous associations, influenced by diet, kidney function, and microbiota composition. These discrepancies highlight the need for longitudinal human studies and interventional trials targeting TMAO metabolism [50,51,52,53,54,55,56,57,58].

#### 3.1.3. Short-Chain Fatty Acids (SCFAs)

Short-chain fatty acids (SCFAs), primarily acetate, propionate, and butyrate, are fermentation products of dietary fibers and exert diverse effects on host metabolism and vascular health. Through activation of G-protein coupled receptors (GPR41/43) and inhibition of histone deacetylases, SCFAs modulate inflammation, lipid metabolism, and endothelial function. Butyrate, in particular, enhances intestinal barrier integrity and reduces systemic inflammation, whereas acetate and propionate display context-dependent actions on lipid and glucose metabolism [5,59,60,61,62,63,64].

SCFAs, mainly acetate, propionate, and butyrate, exert immunomodulatory and vasoprotective effects. Butyrate promotes colonic regulatory T cell (Treg) differentiation via histone deacetylase inhibition [65]. Propionate supplementation reduced hypertension-induced cardiovascular damage and vascular remodeling in animal models, with early clinical data supporting blood pressure reduction [66]. Collectively, SCFAs highlight the potential for microbiota-derived metabolites to act as protective mediators against cardiovascular disease.

While experimental evidence consistently supports protective effects against atherosclerosis, human studies remain limited and heterogeneous, with diet, microbiota composition, and interindividual variability influencing outcomes. Thus, SCFAs represent promising but incompletely validated mediators of cardiometabolic protection [67,68,69].

#### 3.1.4. Secondary Bile Acids

Gut microbiota convert primary bile acids into secondary bile acids (e.g., deoxycholic acid, lithocholic acid), which interact with nuclear and membrane receptors such as the Farnesoid X receptor (FXR) and Takeda G protein-coupled receptor (TGR5). These pathways regulate cholesterol metabolism, glucose homeostasis, and inflammation. Experimental studies suggest dual roles: FXR activation may reduce triglyceride accumulation and vascular inflammation, while excessive secondary bile acids can promote oxidative stress and endothelial injury [46,70,71,72,73,74,75,76,77,78,79,80,81].

Secondary bile acids signal through FXR and TGR5 receptors, influencing lipid metabolism and vascular inflammation. FXR activation reduces triglyceride accumulation and suppresses inflammatory gene expression [82]. TGR5 activation enhances endothelial nitric oxide synthesis, improving vascular tone and energy balance [83]. Conversely, excessive deoxycholic acid has been linked to oxidative stress and vascular injury, suggesting a dual, context-dependent role of bile acids in cardiovascular disease.

Clinical data remain scarce and sometimes contradictory, with variability in bile acid profiles across individuals and disease states. Further studies are required to clarify whether targeting bile acid signaling can provide consistent therapeutic benefit in atherosclerosis [84,85].

**Figure 3:**
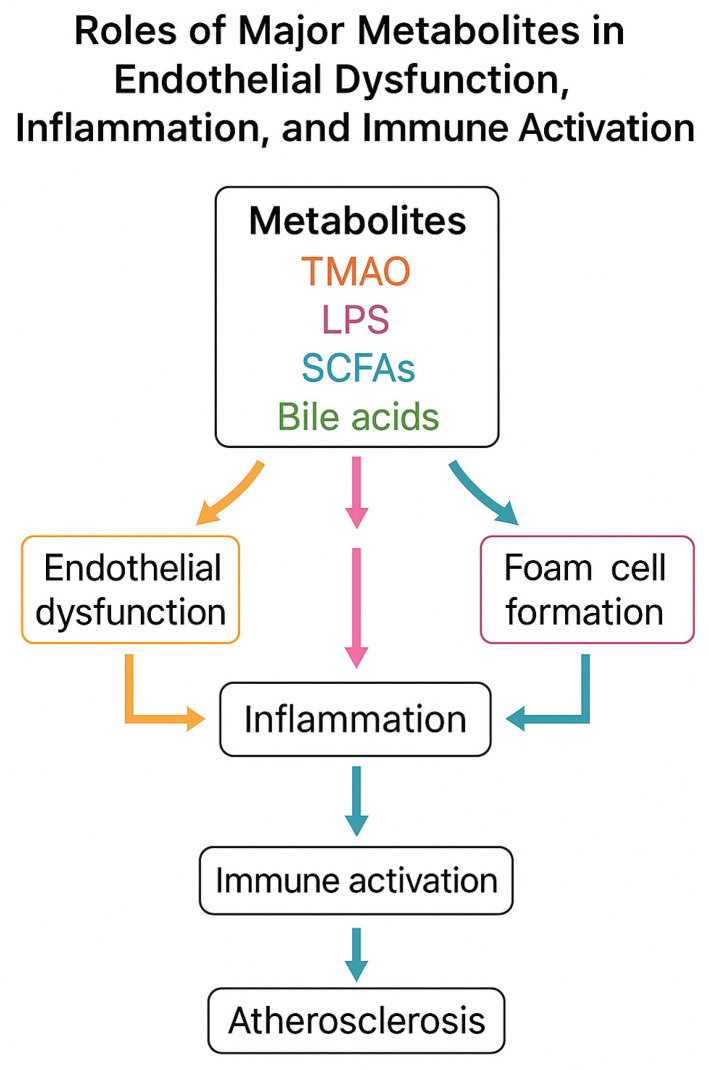
Mechanistic flowchart of gut microbiota-derived metabolites in atherosclerosis. TMAO promotes foam cell formation and endothelial inflammation; LPS activates TLR4/NF-κB signaling and cytokine release; SCFAs induce anti-inflammatory effects and barrier protection; secondary bile acids act via FXR/TGR5 with dual effects. All converge to endothelial dysfunction, inflammation, and plaque formation.

To provide a comprehensive overview of the mechanistic links between gut microbiota-derived metabolites and atherosclerosis, we include a schematic flowchart (Figure 3). This illustration summarizes the main pathways through which TMAO, LPS, SCFAs, and secondary bile acids influence endothelial dysfunction, immune activation, and plaque development.

#### 3.1.5. Emerging and Understudied Microbial Metabolites

In addition to the well-characterized gut microbiota-derived metabolites such as TMAO, LPS, and SCFAs, several emerging metabolites have recently attracted attention for their potential roles in atherosclerosis. Among these, indole derivatives, produced by bacterial metabolism of tryptophan, show diverse and sometimes opposing vascular effects. Indoxyl sulfate, a protein-bound uremic toxin derived from indole metabolism, has been shown to promote oxidative stress, endothelial dysfunction, vascular smooth muscle proliferation, and inflammatory signaling, thereby contributing to vascular injury and atherosclerotic progression [86]. As summarized in Figure 2, indoxyl sulfate exerts pro-atherogenic actions by inducing oxidative stress and endothelial dysfunction. In contrast, indole-3-propionic acid (IPA) and indole-3-aldehyde (IAld) exhibit antioxidant and anti-inflammatory effects; IPA, in particular, has been associated with improved intestinal barrier function and reduced systemic inflammation, while IAld activates the aryl hydrocarbon receptor (AhR), enhancing mucosal immunity and potentially conferring protection against vascular inflammation [87].

**Figure 4:**
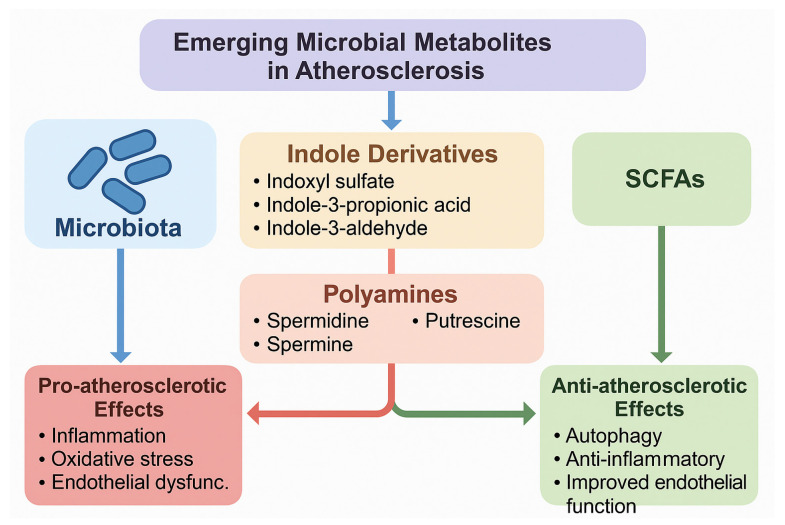
Emerging and understudied gut microbiota-derived metabolites in atherosclerosis. The figure illustrates indole derivatives (indoxyl sulfate, indole-3-propionic acid, indole-3-aldehyde) and polyamines (spermidine, spermine, putrescine) as novel players in vascular homeostasis. Pro-atherosclerotic metabolites (e.g., indoxyl sulfate, putrescine) are associated with oxidative stress, inflammation, and endothelial dysfunction, while protective metabolites (e.g., indole-3-propionic acid, spermidine) exhibit antioxidant, anti-inflammatory, and autophagy-inducing effects.

Another important but understudied group comprises the polyamines, including spermidine, spermine, and putrescine, which are either synthesized directly by gut bacteria or modulated by microbial metabolism. Polyamines are involved in cellular growth, apoptosis, and immune modulation, and recent studies suggest they exert cardioprotective actions through the induction of autophagy, improved mitochondrial function, and suppression of inflammatory pathways [88]. Notably, dietary spermidine supplementation has been linked to extended lifespan, reduced arterial stiffness, and improved endothelial function in animal models, with preliminary evidence of cardiovascular benefits also observed in human cohorts [89]. Despite these promising findings, the mechanistic roles of indole derivatives and polyamines in human atherosclerosis remain incompletely defined, and their dual or context-dependent effects highlight the need for more targeted mechanistic and clinical studies. A schematic representation of these emerging metabolites and their pro- and anti-atherogenic effects is shown in Figure 4.

Table 1 summarizes the key gut microbiota-derived metabolites, their sources, mechanisms, and effects on atherosclerosis, highlighting their dual roles in disease progression.

### 3.2. Biomarkers and Clinical Evidence

Beyond mechanistic insights, several gut microbiota-derived metabolites and microbial signatures have been investigated as potential biomarkers for atherosclerosis and cardiovascular risk. Among these, TMAO has emerged as the most widely studied circulating metabolite, while other candidates such as LPS, SCFAs, and secondary bile acids remain less validated. In parallel, alterations in gut microbiota composition have been proposed as disease-associated signatures. However, significant methodological and interindividual variability limits their clinical utility. This section provides an overview of circulating metabolites and microbiota signatures as biomarkers, and discusses the current evidence and limitations.

#### 3.2.1. Circulating Microbial Metabolites as Biomarkers

- Trimethylamine-N-oxide (TMAO)

Among gut microbiota-derived metabolites, TMAO is the most extensively studied candidate biomarker. Elevated plasma TMAO levels were first linked to adverse cardiovascular outcomes in coronary artery disease patients [52]. Subsequent prospective studies confirmed associations with myocardial infarction, stroke, and mortality, positioning TMAO as a potential independent risk factor. Mechanistically, TMAO promotes foam cell formation, platelet activation, and vascular inflammation, which supports its biomarker potential. Subsequent large prospective cohorts confirmed its predictive value for cardiovascular events and mortality across diverse populations [47,48,49]. A recent integrative study highlighted its role in microbiota–immune crosstalk and vascular inflammation. However, recent large cohorts revealed heterogeneous associations, influenced by renal function, diet, and gut microbiota composition, underscoring the need for standardized cut-off values before clinical translation [46].

- Lipopolysaccharides (LPS)

Circulating endotoxin activity (a proxy for LPS translocation) has been associated with insulin resistance, metabolic syndrome, and higher cardiovascular risk. LPS induces systemic inflammation via TLR4/NF-κB and endothelial dysfunction, mechanistically linking it to atherosclerosis. Yet, its utility as a biomarker is limited by low sensitivity, variability in measurement assays, and confounding by obesity and diabetes. Thus, LPS may serve more as a risk enhancer rather than a stand-alone biomarker [95,96].

- Short-chain fatty acids (SCFAs)

SCFAs such as acetate, propionate, and butyrate are detectable in plasma and stool, reflecting microbial fermentation of dietary fibers. Higher circulating SCFA levels correlate with reduced inflammation and improved metabolic profiles [67]. Propionate supplementation in humans lowered blood pressure and inflammatory markers, suggesting that SCFAs may function as protective biomarkers. However, interindividual variability and limited cardiovascular endpoint studies restrict their clinical applicability [97].

- Secondary bile acids

Alterations in bile acid profiles have been linked with dyslipidemia, insulin resistance, and vascular dysfunction. Secondary bile acids interact with FXR and TGR5, modulating cholesterol and glucose metabolism. Elevated plasma deoxycholic acid has been associated with vascular oxidative stress, while certain bile acid derivatives may exert anti-inflammatory effects. Despite mechanistic plausibility, human studies remain scarce and inconsistent, and bile acids are not yet validated as cardiovascular biomarkers [74,98].

A comparative summary of gut microbiota-derived metabolites and microbial signatures investigated as potential biomarkers for atherosclerosis is provided in Table 2.

#### 3.2.2. Microbiota Signatures in Atherosclerosis

The human gut microbiota, composed of ~100 trillion microorganisms (bacteria, viruses, fungi, parasites), begins colonization at birth and is shaped by delivery mode, maternal microbiota, age, genetics, geography, and especially diet. In healthy individuals, Firmicutes and Bacteroidetes predominate, with key genera such as *Lactobacillus*, *Clostridium*, and *Bifidobacterium*. A healthy microbiota shows high diversity, supporting barrier integrity, immune maturation, nutrient metabolism, and drug absorption [10].

Diet plays a central role: fiber-rich diets promote beneficial microbes (*Bifidobacterium*, *Prevotella*), while Western diets favor bile-tolerant, pro-inflammatory species (*Bilophila*, *Bacteroides*) at the expense of protective taxa (*Roseburia*). Dysbiosis, with reduced diversity and an altered Firmicutes/Bacteroidetes ratio, is common in obesity, often accompanied by decreased *Akkermansia muciniphila*, whose supplementation improves barrier integrity and inflammation [99,100].

Therapeutically, dietary interventions rich in fiber and anti-inflammatory components improve metabolic and inflammatory conditions. Moreover, fecal microbiota transplantation combined with an anti-inflammatory diet has shown superior efficacy in ulcerative colitis, underscoring the microbiome’s therapeutic potential [101].

The gut microbiota plays a central role in nutrient metabolism and host health by transforming carbohydrates, proteins, and fats into metabolites that act locally and systemically. These include beneficial compounds such as short-chain fatty acids (SCFAs), as well as potentially harmful metabolites, depending on substrate type and microbial composition [102,103].

Bacteria dominate microbial metabolism, particularly in the colon, where fermentation produces SCFAs, B vitamins, and biotransformed polyphenols. These processes support colonocyte function, immune regulation, and barrier integrity. However, protein and fat fermentation may also yield toxic products that promote inflammation and epithelial injury [104].

Microbiota-derived metabolites influence distant organs via the gut–brain, gut–liver, gut–kidney, and gut–heart axes, with SCFAs being the best studied. While their anti-inflammatory effects are well established, the impact of protein and lipid metabolites on cardiovascular and metabolic disease remains less clear [105].

The gut microbiome’s extensive genetic repertoire (≈3 million genes vs. ≈23,000 human genes) greatly expands host metabolic capacity, enabling functions that are absent in humans, such as polysaccharide degradation and vitamin synthesis [106]. Evidence from germ-free models and human studies confirms its role in immunity, energy balance, and disease susceptibility, including obesity, diabetes, IBD, and colon cancer [107].

The microbiota also shapes mucosal and systemic immunity: SCFAs promote regulatory T cells (Tregs) via GPCR signaling and epigenetic regulation, while segmented filamentous bacteria induce Th17 differentiation. Beneficial taxa such as *Bifidobacterium* enhance tolerance and reduce inflammation [59,108,109]. Early colonization is critical for intestinal barrier maturation and immune homeostasis, involving epithelial cells, antimicrobial peptides, and gut-associated lymphoid tissue [110,111]. This lifelong crosstalk between microbes and host immunity underscores the therapeutic potential of microbiota-targeted strategies [112].

In health, the gut microbiota (~10^14^ cells, >1000 species, mainly Firmicutes and Bacteroidetes) maintains homeostasis and prevents pathogenic overgrowth. Dysbiosis may involve loss of beneficial microbes, expansion of harmful species, and reduced diversity, often occurring together. It has been associated with multiple diseases, including cardiovascular disease, IBD, obesity, diabetes, allergies, autism, and colorectal cancer [113].

Advances in microbiome research have enabled strategies to counteract dysbiosis, ranging from diet and antibiotics to probiotics, fecal microbiota transplantation (FMT), and environmental interventions. These approaches aim to restore microbial balance, strengthen barrier integrity, and modulate immunity. Future studies must integrate genetic, dietary, and environmental factors to develop targeted, personalized microbiota-based therapies [114].

Table 3 compares the gut microbiota composition in healthy versus dysbiotic states, illustrating microbial shifts linked to atherosclerosis.

#### 3.2.3. Limitations and Variability in Biomarker Interpretation

Despite growing interest in gut microbiota-derived metabolites as cardiovascular biomarkers, several limitations hinder their clinical translation, as detailed below.

Interindividual variability. Microbiota composition is highly individual, influenced by genetics, age, diet, geography, medication use (antibiotics, statins), and comorbidities. Consequently, the same metabolite (e.g., TMAO) may show divergent associations with disease risk across populations [46,52].

Dietary and environmental confounders. Nutrient intake directly shapes microbial metabolites, making it difficult to disentangle diet-derived effects from microbiota-driven variability. For instance, red meat consumption strongly affects plasma TMAO levels, potentially confounding its predictive value [46,52,115,116].

Methodological heterogeneity. Biomarker studies often use different analytical techniques (16S rRNA sequencing vs. shotgun metagenomics for microbiota; ELISA vs. mass spectrometry for metabolites), limiting comparability. Lack of standardized assays and cut-off values hampers reproducibility across cohorts [117,118,119,120,121,122].

Functional redundancy. Multiple microbial taxa can produce the same metabolite, while a single species may contribute to multiple pathways. Thus, composition alone cannot predict function, and metabolite concentrations may not directly reflect biological effects [123].

Dynamic and context-dependent effects. Many microbial metabolites (e.g., SCFAs, secondary bile acids) have dual roles: they are protective in some contexts, and harmful in others. Without longitudinal and functional studies, cross-sectional measurements risk misinterpretation [124].

Limited clinical validation. While TMAO has robust epidemiological support, most other metabolites lack prospective or interventional studies. Translation into routine practice requires large, standardized, longitudinal trials assessing their incremental value beyond established risk factors [46].

Current evidence highlights promising microbiota-derived biomarkers, but their clinical use is constrained by variability, methodological inconsistencies, and lack of standardization. Future research should adopt integrated multi-omics approaches, harmonized protocols, and large-scale prospective studies to define reliable, clinically actionable microbiota-based biomarkers.

### 3.3. Therapeutic Interventions Targeting Microbiota

The gut microbiome plays a critical role in modulating cardiometabolic risk by acting as a sensor, modulator, and translator of host metabolic changes through its metabolites, including SCFAs, TMAO, secondary bile acids, and phenylacetylglutamine.

Diet is a major determinant of both microbiome composition and cardiometabolic health, with dietary interventions, especially those rich in fiber, shown to influence microbial activity and metabolite production. Clinical studies indicate that over 70% of interventions targeting the gut microbiome result in significant improvements in cardiometabolic traits (such as obesity and type 2 diabetes), although only 63% report changes in microbiome composition [84].

Prebiotic interventions most consistently alter microbiota, followed by dietary changes, while probiotics show the least impact, partly due to poor colonization efficiency. No significant difference in efficacy was observed between single- and multi-strain probiotics or synbiotics. Notably, dietary interventions outperformed probiotics in achieving both clinical benefits and microbiome shifts, suggesting that whole-diet strategies may better modulate complex microbial communities. While microbial changes appear to mediate clinical improvements in many studies, methodological inconsistencies hinder definitive conclusions [125,126].

The evidence supports the therapeutic modulation of the gut microbiome to improve cardiometabolic outcomes, yet highlights the need for large, well-controlled trials with standardized biomarkers to identify mechanistic pathways and establish evidence-based guidelines for microbiome-targeted therapies [127].

#### 3.3.1. Diet

Diet is a major determinant of gut microbiota composition and function, capable of modulating microbial diversity, interactions, and resilience. Long-term consumption of plant-based, fiber-rich diets has been shown to enhance SCFA production, strengthen mucosal barriers, and increase beneficial bacteria such as Actinobacteria and Bacteroidetes. Interventions with Mediterranean diets can rapidly shift microbial profiles toward health-promoting genera like *Butyricicoccus* and *Roseburia*, while less healthy diets (Canadian-style) favor potentially dysbiotic taxa. Polyphenol- and bioactive-enriched diets, including green tea and orange juice, also enhance microbiota diversity and support SCFA-producing microbes [99,128].

Animal studies highlight that dietary timing is critical: pre-, during, and post-antibiotic consumption of fiber-rich foods (oats) better preserves microbial diversity and mitigates dysbiosis. Similarly, high-fiber diets in humans enhance post-antibiotic microbiome recovery and reduce the abundance of antibiotic resistance genes. Vegan and omnivorous diets that are rich in fermentable fibers increase Firmicutes abundance and butyrate production, contributing to microbial resilience [129]. However, despite these promising findings, the precise mechanisms and key dietary drivers that promote long-term microbiome stability and recovery remain poorly understood, limiting targeted nutritional strategies for microbiome restoration [130].

It was demonstrated that an high-fat diet (HFD), particularly its low fiber content, induces gut microbiota dysbiosis, reducing SCFA production and impairing intestinal immune defenses. This promotes systemic inflammation and atherosclerosis by enhancing gut-derived immune cell trafficking to vascular sites. FMT experiments establish a causal link between HFD-shaped microbiota and atherosclerosis, independent of metabolic alterations. Fiber supplementation (with fructooligosaccharide) mitigates these effects by restoring microbial balance and SCFA levels. These findings underscore dietary fiber’s therapeutic potential in preventing microbiota-driven CVDs and highlight the gut–immune–vascular axis as a critical pathway in atherosclerosis [131,132].

A recent study in a Southeast Asian population linked healthy plant-based diets rich in dietary fiber to reduced cardiometabolic risk, unlike unhealthy plant-based foods (refined grains, fried snacks, sugar-sweetened beverages). Hutchison et al. demonstrated that dietary fiber attenuates atherosclerosis through gut microbiome modulation, while Sakurai et al. showed that alpha-cyclodextrin, a glucose-based cyclic polymer, reduces atherosclerosis by altering cecal bacteria composition. Additional studies confirm that high intakes of fibers like inulin and pectin similarly mitigate atherosclerosis, suggesting that dietary fiber plays a critical role in halting the onset and progression of atherosclerosis via microbiome-mediated mechanisms [26,133].

#### 3.3.2. Prebiotics

Strengthening the stability and resilience of the gut microbiome may be achieved through synbiotic interventions, which combine live microorganisms with substrates selectively utilized by host microbes. While prebiotics and probiotics can increase the abundance of beneficial bacteria such as *Bifidobacterium* and *Lactobacillus*, their effects on overall microbiota composition are often modest and usually persist only during the intervention [134].

The type, dosage, and duration of prebiotic intake influence the enrichment of specific bacterial groups. Soluble fibers like pectin and inulin are fermented by beneficial microbes to produce SCFAs, which acidify the colon, inhibit pathogens, and contribute to immune modulation, epithelial barrier function, and metabolic health. Insoluble fibers like cellulose and lignin support *Prevotella* and *Ruminococcus*, leading to other anti-inflammatory metabolites [135].

Novel prebiotics, such as bifidobacterial-galacto-oligosaccharides (B-GOS), show promise in reducing travelers’ diarrhea, while polysaccharides from the mushroom *Dictyophora indusiata* (DIP) have demonstrated the ability to restore microbiota after antibiotic-induced dysbiosis and reduce inflammation. DIP promotes beneficial bacterial families and SCFA production while suppressing harmful taxa, as confirmed by both in vivo mouse studies and in vitro fermentation assays. These findings underscore the potential of targeted synbiotic and fiber-based strategies to foster a resilient, health-promoting gut microbiome [136].

Recent studies underscore the therapeutic potential of polysaccharides/oligosaccharides, particularly when combined with zinc, in addressing obesity-related metabolic disorders and atherosclerosis through gut microbiome modulation. Research indicates that obese individuals have lower zinc levels, linked to metabolic disorders, and zinc supplementation may mitigate obesity. A novel Ulva oligosaccharide-based zinc supplement was shown to improve intestinal flora, reduced dyslipidemia, and lowered body weight in obese mice. Additionally, mannose oligosaccharides were found to prevent atherosclerosis progression by lowering serum cholesterol, increasing cecal butyrate, and enhancing bile acid excretion via microbiome changes. Moreover, *Laminaria japonica* polysaccharides suppressed atherosclerosis by boosting autophagy pathways, while red algal polysaccharides are emerging as a potential treatment. These findings highlight innovative, microbiome-mediated strategies using prebiotic polysaccharides and zinc supplementation for preventing and treating obesity and atherosclerosis [137,138].

#### 3.3.3. Probiotics

Probiotics support gut health by competing with pathogens, enhancing mucosal integrity, modulating immunity, and producing beneficial metabolites and antimicrobial peptides. However, clinical evidence on their role in restoring the human gut microbiome (HGM) post-antibiotics or enhancing its resilience remains inconsistent. Some studies suggest that probiotics may delay microbiome recovery due to competition with native taxa and immune activation, while others report benefits such as reduced antibiotic-associated diarrhea and increased SCFA production [139].

These conflicting results highlight the complexity of host–microbe interactions and the limitations of current probiotic strains. Meta-analyses show limited evidence supporting the role of probiotics in preventing travelers’ diarrhea, with only *S. boulardii* showing consistent efficacy. Most clinical interventions have focused on co- or post-treatment administration, with few exploring pre-antibiotic probiotic use. Some studies show modest improvement in microbial diversity, while others, including Suez et al. (2018) [139], report delayed recovery and increased inflammation with multi-strain probiotic blends [140].

New approaches propose assessing functional rather than compositional shifts and exploring next-generation probiotics such as *B. uniformis*, *A. muciniphila*, and *F. prausnitzii*, which have shown promise in preclinical models for restoring microbiota, reducing inflammation, and promoting mucosal healing. Synergistic combinations of *Lactobacillus*, *Bifidobacterium*, *Bacteroides*, and *Akkermansia* strains demonstrate superior recovery outcomes in antibiotic-treated mice compared to single-strain therapies. These findings underscore the potential of advanced probiotic formulations and functional assessment metrics in developing effective strategies for gut microbiome resilience and recovery [141].

Probiotics, live microorganisms found in fermented foods like yogurt, kefir, and sauerkraut, confer health benefits by modulating gut microbiota, lipid profiles, endothelial function, oxidative stress, and inflammation, offering potential for atherosclerosis (AS) prevention and treatment. Strains like *Lactobacillus* and *Bifidobacterium* reduce AS risk by lowering cholesterol, trimethylamine N-oxide (TMAO), and inflammatory markers while enhancing gut barrier integrity and short-chain fatty acid (SCFA) production. In ApoE−/− mice, *Lactiplantibacillus plantarum* ATCC 14,917 (10^9^ CFU, 12 weeks) prevented plaque formation by improving intestinal integrity, while multi-strain probiotics reduced vascular inflammation. *L. plantarum* ZDY01 lowered TMAO in choline-fed mice, and *L. reuteri* and *Bifidobacterium* species increased fecal SCFAs, correlating with reduced hepatic cholesterol [142,143].

Human trials show that *Lactobacillus* and *Bifidobacterium* strains improve HDL, lower total cholesterol, and enhance endothelial function in coronary artery disease patients. Probiotics also reduce oxidative stress (lower ox-LDL, MDA) and inflammation (reduced IL-1, TNFα) by inhibiting NF-κB signaling. Emerging *Lactobacillus fermentum* strains combined with phenolic compounds like quercetin and resveratrol show enhanced antioxidant and anti-inflammatory effects, suggesting novel nutraceutical potential. However, optimal strain combinations, treatment duration, and long-term safety require further clinical validation [144,145,146].

Targeting gut microbiota through dietary fiber supplementation, probiotics, or prebiotics could reduce systemic inflammation and atherosclerosis risk. Further research into personalized microbiota interventions and immune cell trafficking mechanisms may enhance CVD prevention strategies [14].

#### 3.3.4. Small Molecule Compounds

Recent research highlights innovative methods for regulating the gut microbiome to mitigate atherosclerosis, focusing on probiotics, prebiotics, and small molecules. While probiotics and prebiotics are widely used, their safety, colonization stability, and precise control over microbial composition remain challenging. Scientists at Scripps Research Institute developed a screening method to identify d- and L-α-peptide cyclic molecules that selectively inhibit bacterial growth by disrupting cell membrane function.

In high-fat diet-induced atherosclerotic mice, these peptides reduced cholesterol by 36% after 2 weeks and atherosclerotic plaque area by ~40% after 10 weeks, shifting gut microbiota toward a low-fat diet profile. Additionally, Wang et al. showed that 3,3-dimethyl-1-butanol, a choline analog, attenuated atherosclerosis by inhibiting microbial TMA-lyase activity and TMA/TMAO production. A Ganoderma meroterpene derivative was identified that enriched *Parabacteroides merdae*, enhancing branched-chain amino acid catabolism and improving obesity-related atherosclerosis. These findings reveal novel microbiota-mediated mechanisms of atherosclerosis progression and propose targeted small-molecule interventions for cardiovascular health improvement [26,57,147,148].

#### 3.3.5. Phenolic Compounds

Phenolic compounds, particularly polyphenols like quercetin, resveratrol, curcumin, gallic acid, naringin, procyanidin, geraniin, and protocatechuic acid, show significant potential in preventing and treating atherosclerosis (AS) by modulating gut microbiota and related metabolic pathways [149].

Quercetin, found in foods like apples and red wine, reduces atherosclerotic lesions, cholesterol, and inflammatory markers (TNF-α, IL-6) in ApoE−/− and LDLr−/− mice by increasing gut microbial diversity and beneficial genera like *Akkermansia* and *Bacteroides*, while altering bile acid and coprostanol metabolism. Resveratrol, abundant in grapes and berries, lowers TMAO, cholesterol, and LDL, enhances HDL, and improves endothelial function via eNOS and PKA signaling, with gut microbiota modulation increasing *Bacteroides* and *Lactobacillus* [150].

Curcumin restores the Firmicutes/Bacteroidetes ratio and reduces TMAO and LPS levels, while gallic acid shows sex-specific reductions in plaque formation. Naringin enhances bile acid excretion and shifts microbial composition, favoring *Lactobacillus* and reducing TMA-producing bacteria. Procyanidin A2 and geraniin increase microbial diversity and beneficial genera like *Akkermansia*, reducing plaque area, while protocatechuic acid decreases inflammation without affecting TMAO [151].

These compounds collectively improve gut barrier integrity, reduce pro-atherogenic metabolites (TMAO, LPS), and enhance anti-inflammatory and antioxidant effects, suggesting that dietary intake of polyphenol-rich foods could be a promising strategy for AS prevention and treatment, although further studies are needed to explore synergistic effects with probiotics and precise mechanisms [152].

#### 3.3.6. Targeting TMAO

Trimethylamine N-oxide (TMAO), a gut microbiota-derived metabolite, is a recognized risk factor for atherosclerosis (AS), prompting research into strategies to reduce its levels through dietary interventions, gut flora regulation, inhibition of TMAO precursor production, fecal microbiota transplantation (FMT), and pharmacological approaches, including traditional Chinese medicine [153].

Dietary interventions like the Mediterranean diet, vegan diets, and intermittent fasting lower TMAO by modulating gut microbiota, with studies showing reduced TMAO in vegetarians and those on fasting-mimicking diets due to shifts in microbial composition (lower *Clostridia*, higher *Trichospira*). However, nutrients like choline and L-carnitine, while essential, can increase TMAO, necessitating a balance to retain their cardiovascular benefits. Regulating intestinal flora with antibiotics suppresses TMAO but risks dysbiosis and resistance, while probiotics like *Lactobacillus plantarum* ZDY04 and *Bifidobacterium* species reduce TMAO and improve lipid metabolism, although results vary by strain [154].

Inhibiting TMAO precursor production targets microbial choline TMA lyase (CutC) with inhibitors like 3,3-dimethyl-1-butanol (DMB), iodomethylcholine (IMC), and fluoromethylcholine (FMC), which reduce TMAO and plaque formation without harming gut flora. Inhibiting TMA conversion to TMAO by targeting flavin-containing monooxygenase 3 (FMO3) with compounds like trigonelline or antisense oligonucleotides decreases TMAO and thrombosis risk, although safe inhibitors are needed [52]. The structural analogue 3,3-dimethyl-1-butanol (DMB) was first shown to inhibit TMA lyases and reduce atherosclerosis in mice [57]. More recently, Roberts et al. developed a mechanism-based inhibitor, iodomethylcholine, that irreversibly targets microbial CutC/D enzymes, suppresses TMA formation, and attenuates atherosclerosis without altering gut microbial viability [155].

FMT shows potential to alter microbiota and reduce TMAO, but limited studies and ethical concerns restrict its use. Pharmacological approaches, including metformin and resveratrol, modulate gut microbiota and lower TMAO, while traditional Chinese medicines like berberine and Ganoderma reduce TMAO by adjusting microbial composition and inhibiting TMA synthesis, showing promise but requiring further clinical validation. These strategies highlight the potential of targeting TMAO to mitigate AS, with ongoing research needed to optimize efficacy and safety [156].

#### 3.3.7. Fecal Microbiota Transplantation

Fecal microbiota transplantation (FMT), a method involving the transfer of filtered fecal samples from healthy donors or autologous sources to restore gut microbiota balance, has gained traction as a therapeutic approach for dysbiosis-related conditions, including atherosclerosis. Initially developed in the 1960s, FMT is highly effective for treating *Clostridium difficile* infections and shows promise in extra-intestinal diseases like cardiovascular diseases (CVDs) by altering bile acid composition and reducing plasma triglycerides, as seen in obese recipients receiving FMT from lean donors [116,157].

These changes suggest anti-atherosclerotic effects by mitigating dyslipidemia and pro-atherogenic metabolites like trimethylamine N-oxide (TMAO). Preclinical studies demonstrate that FMT from healthy donors reduces plaque development and systemic inflammation in atherosclerosis models. However, human evidence is limited, with small-scale studies showing microbiota shifts but no significant improvements in TMAO, lipids, or endothelial function. One ongoing clinical trial (NCT04410003) is investigating FMT for severe atherosclerosis, but larger trials are needed to confirm efficacy. Risks, including endotoxin transfer and long-term safety concerns, currently limit FMT’s widespread use, necessitating further research to optimize its therapeutic potential in atherosclerosis management [158,159,160].

Table 4 summarizes therapeutic interventions targeting the gut microbiota, including their mechanisms, evidence from studies, and current challenges.

While preclinical models consistently show that microbiota-targeted interventions such as probiotics, prebiotics, and fecal microbiota transplantation (FMT) attenuate atherosclerosis, the clinical applicability remains uncertain, as detailed below.

Probiotics and prebiotics: Some randomized controlled trials suggest modest benefits. For instance, supplementation with *Lactobacillus plantarum* reduced LDL-C and inflammatory markers in hypercholesterolemic patients [161], whereas in other cohorts effects were minimal [162]. Prebiotic fibers such as inulin improved lipid and glucose metabolism and lowered TMAO levels in overweight adults [164], although interindividual variability remained high.

FMT: Although FMT reshapes gut microbiota composition, cardiovascular endpoints have not yet been systematically evaluated in humans. A pilot randomized trial in obese men [163] demonstrated safety and improvements in insulin sensitivity, but cardiovascular outcomes were not assessed.

Dietary interventions: The Mediterranean diet, rich in fibers and polyphenols, lowers circulating TMAO and increases SCFA production. In the landmark PREDIMED trial (>7000 participants), adherence to a Mediterranean diet reduced major cardiovascular events and was associated with favorable shifts in microbiota-derived metabolites [95].

Collectively, these findings underscore that microbiota-based therapies are promising but remain in an early translational phase, with the need for well-powered, long-term randomized trials in cardiovascular populations. These findings are summarized in Table 5, which highlights both the modest benefits observed in small-scale RCTs and the robust evidence from large dietary intervention trials.

Ongoing clinical trials investigating microbiota-targeted strategies are summarized in Table 6. Despite promising results in preclinical and early clinical studies, translation into cardiovascular practice remains challenging. Several ongoing studies have been identified in ClinicalTrials.gov, highlighting the growing interest in microbiota-targeted strategies for cardiometabolic disease.

**Figure 5:**
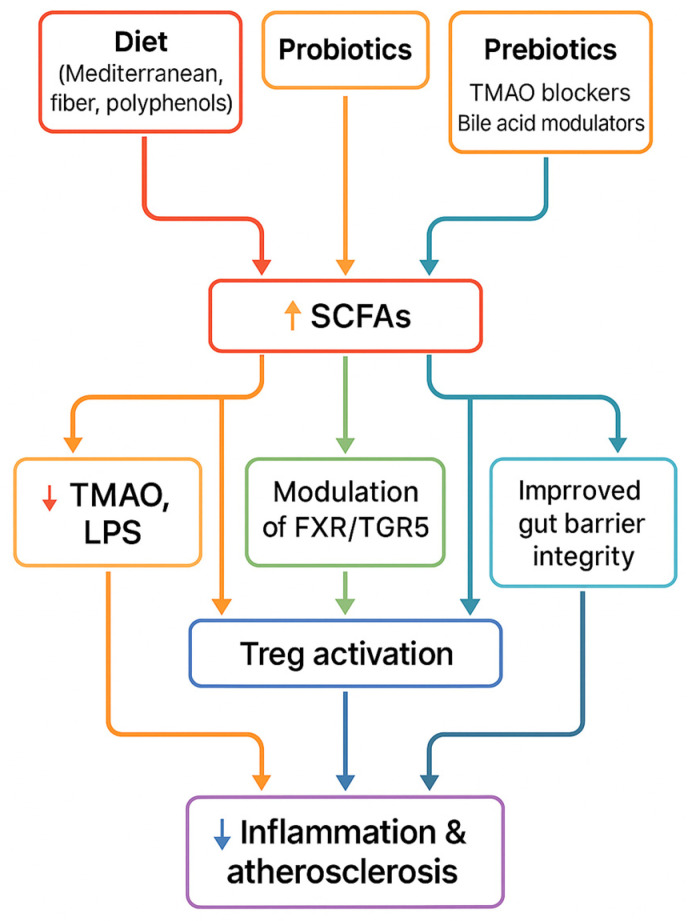
Therapeutic strategies (diet, probiotics, prebiotics, FMT, pharmacological inhibitors) converge on shared mechanisms—↑ SCFAs, ↓ TMAO, improved barrier integrity, immune modulation—leading to reduced inflammation and atherosclerosis.

To summarize the therapeutic section, Figure 5 illustrates how dietary, microbial, and pharmacological interventions converge on common pathways—including increased SCFA production, reduced TMAO, improved gut barrier function, and immune modulation—that ultimately lead to reduced vascular inflammation and atherosclerosis.

## 4. Conclusions and Future Directions

This review consolidates current evidence highlighting the gut microbiota as a central player in atherosclerosis, a multifaceted cardiovascular disease with mechanisms still under exploration. Through the production of metabolites such as trimethylamine N-oxide (TMAO), lipopolysaccharides (LPS), short-chain fatty acids (SCFAs), and bile acids, the gut microbiota contributes to vascular inflammation, endothelial dysfunction, and immune modulation. These microbial products not only provide mechanistic insight but also hold promise as diagnostic biomarkers and therapeutic targets.

Future research must move beyond association and address causality and clinical translation. Large-scale longitudinal and interventional studies are needed to validate microbiota-derived metabolites as biomarkers of cardiovascular risk. The integration of multi-omics technologies (metagenomics, metabolomics, transcriptomics) with clinical phenotyping may help identify robust microbial signatures. A major frontier lies in the development of personalized microbiota-based therapies, including tailored dietary interventions, targeted pre/probiotics, and fecal microbiota transplantation adapted to patient-specific microbial and genetic profiles. Novel pharmacological strategies targeting microbial metabolism, such as TMAO inhibitors or bile acid receptor agonists, also warrant investigation in early-phase clinical trials. 

Despite major advances, significant knowledge gaps remain. First, while inhibition of TMAO production reduces atherosclerosis in preclinical models, it is unknown whether such strategies can reduce plaque burden or cardiovascular events in humans. Second, microbiota-derived biomarkers (TMAO, SCFAs, bile acids) show promise, but their clinical utility is limited by variability in assays, diet, and host genetics, requiring standardization and validation in prospective cohorts. Third, the role of emerging metabolites (e.g., indole derivatives, polyamines) is not fully understood, and their mechanistic contribution to vascular disease requires clarification. Fourth, the interplay between microbiota and the immune system is complex; recent findings suggest causal links, but how immune–microbiota crosstalk translates into human atherosclerosis remains uncertain. Finally, personalization is a major challenge, as diet, genetics, comorbidities, and drug use must be considered when designing therapeutic strategies.

Ultimately, translating microbiota science into cardiovascular medicine will require personalized, mechanistically informed, and clinically validated approaches that integrate microbiome modulation into the broader framework of precision cardiology.

## Figures and Tables

**Table 1:** Key gut microbiota-derived metabolites and their roles in atherosclerosis.

Metabolite	Source/Pathway	Main Effects on Host	References
TMAO	Dietary choline/carnitine → microbial TMA → hepatic FMO3	Foam cell formation, platelet activation, endothelial inflammation	Koeth 2013 [29]; Zhu 2016 [44]; Chen 2017 [56]
LPS	Gram-negative bacteria cell wall	TLR4/NF-κB activation, cytokine release, endothelial dysfunction	Wiedermann 1999 [41]; Miller 2007 [40]
SCFAs	Fermentation of dietary fibers	Tregs induction, anti-inflammatory, barrier protection, ↓ BP	Furusawa 2013 [65]; Bartolomaeus 2019 [66]
Secondary bile acids	Microbial conversion of primary bile acids	FXR/TGR5 signaling, lipid modulation, dual roles	Watanabe 2004 [82]; Pols 2011 [83]
Indoxyl sulfate	Microbial tryptophan metabolism	Endothelial dysfunction, oxidative stress, vascular smooth muscle proliferation	Barreto 2009 [90]; Mutsaers 2015 [91]
Indole-3-propionic acid (IPA)	Tryptophan metabolism by *Clostridium* spp.	Antioxidant, gut barrier protection, anti-inflammatory	Venkatesh 2014 [92]; Konopelski 2019 [93]
Indole-3-aldehyde (IAld)	Tryptophan metabolism → AhR ligand	AhR activation, mucosal immunity, anti-inflammatory	Zelante 2013 [94]
Polyamines (spermidine, spermine)	Microbial amino acid metabolism	Autophagy induction, mitochondrial protection, ↓ arterial stiffness	Eisenberg 2016 [89]; Madeo 2018 [88]

**Table 2:** Gut microbiota-derived metabolites and microbiota signatures investigated as biomarkers in atherosclerosis and cardiovascular disease.

Biomarker/Microbiota Signature	Biological Source	Clinical Evidence	Key Outcomes	References
TMAO (trimethylamine-N-oxide)	Plasma/serum	Multiple large cohorts (Tang 2013 [52] NEJM; subsequent JACC, Circulation studies)	Elevated TMAO predicts MI, stroke, mortality; independent risk factor in CAD and CKD	Tang 2013 [52]; Witkowski 2020 [46]
LPS (endotoxin activity)	Plasma (endotoxin activity assay)	Observational studies in obesity, diabetes, CAD	Associated with low-grade inflammation and endothelial dysfunction; predictive value inconsistent	Pussinen 2011 [95]; Lassenius 2016 [96]
SCFAs (acetate, propionate, butyrate)	Plasma, stool	Small human studies, dietary intervention trials	Higher SCFA levels associated with reduced inflammation, better metabolic profile; limited CV-specific data	Vinolo 2011 [67]; Chambers 2019 [97]
Secondary bile acids (deoxycholic, lithocholic acid)	Plasma, bile	Emerging data; small cohorts	Altered bile acid profiles linked with dyslipidemia, metabolic syndrome, vascular dysfunction	Sayin 2013 [98]; Jia 2018 [74]
Microbiota signatures (diversity, Firmicutes/Bacteroidetes ratio, *Akkermansia*, Enterobacteriaceae)	Stool (16S rRNA, shotgun metagenomics)	Cross-sectional and case-control studies in atherosclerosis, CAD, stroke	Reduced diversity and specific taxa shifts associated with atherosclerosis; not reproducible across cohorts	Jia 2018 [74]; Witkowski 2020 [46]

**Table 3:** Gut microbiota composition in healthy vs. dysbiotic states.

Phyla/Genera	Healthy State	Dysbiotic State (Atherosclerosis)	Impact on Atherosclerosis
Firmicutes	High abundance (e.g., *Lactobacillus*, *Clostridium*)	Increased (elevated F/B ratio)	Promotes inflammation, TMAO production
Bacteroidetes	High abundance (e.g., *Bacteroides*, *Prevotella*)	Decreased	Reduced SCFA production, impaired barrier function
Actinobacteria	Present (e.g., *Bifidobacterium*)	Decreased	Reduced anti-inflammatory effects
Proteobacteria	Low abundance	Increased (e.g., *Escherichia*, *Klebsiella*)	Enhances LPS-mediated inflammation
*Akkermansia muciniphila*	High abundance	Decreased	Impaired gut barrier, increased inflammation

**Table 4:** Therapeutic interventions targeting gut microbiota in atherosclerosis.

Intervention	Mechanism of Action	Main Effects	Clinical Evidence (Human RCTs)	References
Probiotics (*Lactobacillus*, *Bifidobacterium*)	Modulate gut composition, ↓ cholesterol, ↓ inflammation	↓ LDL-C, ↓ CRP (inconsistent results)	Yes (small RCTs, n < 200, modest effects)	Jones 2012 [161]; Horvath 2020 [162]
Prebiotics (inulin, resistant starch)	↑ SCFA production, ↓ TMAO, improved insulin sensitivity	Improved lipid/glucose metabolism	Yes (RCTs in overweight adults)	Chen 2020 [147]
FMT (fecal microbiota transplantation)	Restore microbial balance, ↑ diversity	Improved insulin sensitivity, altered microbiota composition	No (pilot studies only, no CV endpoints)	Kootte 2017 [163]
Mediterranean diet	↑ SCFAs, ↓ TMAO, ↑ polyphenol metabolism	↓ CV events, improved vascular function	Yes (large RCT: PREDIMED > 7000 participants)	Estruch 2018 [6]
TMAO inhibitors (DMB, iodomethylcholine)	Block microbial TMA formation	↓ TMAO, ↓ atherosclerosis in mice	No (preclinical only)	Wang 2015 [57]
Bile acid modulators (FXR, TGR5 agonists)	Regulate lipid metabolism, ↓ inflammation	↓ triglycerides, improved vascular tone	No (mainly animal studies)	Watanabe 2004 [82]; Pols 2011 [83]

**Table 5:** Human evidence for microbiota-targeted therapies in CVD.

Intervention	Population	Study Design	Key Outcomes	References
*Lactobacillus plantarum*	Hypercholesterolemic adults	RCT, 12 weeks	↓ LDL-C, ↓ CRP	Jones et al., 2012 [161]
Multistrain probiotics	Metabolic syndrome	RCT, 8 weeks	No significant CV effects	Horvath et al., 2020 [162]
Inulin (prebiotic)	Overweight adults	RCT, 6 weeks	↓ TMAO, improved insulin sensitivity	Chen et al., 2020 [147]
FMT (lean donor)	Obese men	Pilot RCT	Altered microbiota, ↑ insulin sensitivity, no CV endpoints	Kootte et al., 2017 [163]
Mediterranean diet	At-risk population (PREDIMED trial)	Multicenter RCT, >7000	↓ CV events, lower TMAO, ↑ SCFAs	Estruch et al., 2018 [6]

**Table 6:** Ongoing clinical trials.

Intervention	Condition	Clinical Trial ID	Status	Primary Outcomes	Notes
FMT (from lean donors)	Obesity & metabolic syndrome	NCT04410003	Recruiting	Microbiota composition, insulin sensitivity	No CV endpoints yet
Probiotic (multistrain)	Coronary artery disease	NCT05678932	Recruiting	Lipid profile, inflammatory markers	Focus on LDL-C and CRP
Prebiotic (inulin, resistant starch)	Hypertension	NCT05321882	Active, not recruiting	Blood pressure, SCFA levels	Assessing microbiota–BP link
Mediterranean diet intervention	High-risk CV patients	NCT04899314	Recruiting	CV events, TMAO levels	Extension of PREDIMED
TMAO-lowering agent (3,3-dimethyl-1-butanol, DMB)	Metabolic syndrome	NCT05136840	Recruiting	TMAO levels, endothelial function	First-in-human metabolic trial

## Data Availability

Data are contained within the article.

## Abbreviations

The following abbreviations are used in this manuscript:

*A. muciniphila*	*Akkermansia muciniphila*
ABCA1	ATP-binding cassette transporter A1
ABCG2	ATP-binding cassette super-family G member 2
ACC	Acetyl-CoA carboxylase
Akt	Protein kinase B (also part of PI3K-Akt pathway)
AMPK	AMP-activated protein kinase
ApoA-I	Apolipoprotein A-I
ApoE	Apolipoprotein E
AS	Atherosclerosis
ASBT	Apical sodium-dependent bile acid transporter
ASCVD	Atherosclerotic cardiovascular disease
ATG16L1	Autophagy related 16 like 1
*B. uniformis*	*Bacteroides uniformis*
bai	Bile acid-inducible (operon)
BARs	Bile acid receptors
BAs	Bile acids
B-GOS	Bifidobacterial-galacto-oligosaccharides
BSH	Bile salt hydrolase
CA	Cholic acid
CAR	Constitutive androstane receptor
CD14	Cluster of differentiation 14
CD204	Cluster of differentiation 204
CD36	Cluster of differentiation 36
CDCA	Chenodeoxycholic acid
COX-2	Cyclooxygenase-2
COVID-19	Coronavirus disease 2019
CRC	Colorectal cancer
CVD	Cardiovascular disease
CYP27A1	Cytochrome P450 family 27 subfamily A member 1
CYP7A1	Cytochrome P450 family 7 subfamily A member 1
CYP7B1	Cytochrome P450 family 7 subfamily B member 1
DCA	Deoxycholic acid
DCs	Dendritic cells
DIP	*Dictyophora indusiata* polysaccharide
DMB	3,3-dimethyl-1-butanol
*E. coli*	*Escherichia coli*
EDH	Endothelium-derived hyperpolarization
EGFR	Epidermal growth factor receptor
ENS	Enteric nervous system
eNOS	Endothelial nitric oxide synthase
ERK1/2	Extracellular signal-regulated kinase 1/2
*F. prausnitzii*	*Faecalibacterium prausnitzii*
F/B	Firmicutes/Bacteroidetes (ratio)
FASN	Fatty acid synthase
FFAR2	Free fatty acid receptor 2
FFAR3	Free fatty acid receptor 3
FMC	Fluoromethylcholine
FMO3	Flavin monooxygenase 3
FMT	Fecal microbiota transplantation
FOXP3	Forkhead box P3
FXR	Farnesoid X receptor
G6Pase	Glucose-6-phosphatase
GABA	Gamma-aminobutyric acid
GF	Germ-free
GLP-1	Glucagon-like peptide 1
GPCR	G-protein coupled receptor
GPR41	G-protein coupled receptor 41
*H. pylori*	*Helicobacter pylori*
HDAC	Histone deacetylase
HDACs	Histone deacetylases
HDL	High-density lipoprotein
HFD	High-fat diet
HGM	Human gut microbiome
HMG-CoA	3-hydroxy-3-methylglutaryl-coenzyme A
HSDH	Hydroxysteroid dehydrogenase
I3C	Indole-3-carbinol
IBD	Inflammatory bowel disease
IBS	Irritable bowel syndrome
ICAM-1	Intercellular adhesion molecule 1
IEC	Intestinal epithelial cell
IECs	Intestinal epithelial cells
IFIT1	Interferon-induced protein with tetratricopeptide repeats 1
IgA	Immunoglobulin A
IgG	Immunoglobulin G
IL-1	Interleukin-1
IL-1β	Interleukin-1 beta
IL-6	Interleukin-6
IL-8	Interleukin-8
IMC	Iodomethylcholine
isoalloLCA	Isoallolithocholic acid
JNK	c-Jun N-terminal kinase
LAL	Limulus Amoebocyte Lysate
LBP	Lipopolysaccharide-binding protein
LCA	Lithocholic acid
LDL	Low-density lipoprotein
LDLr	Low-density lipoprotein receptor
LL-37	Cathelicidin antimicrobial peptide
LOX-1	Lectin-like oxidized low-density lipoprotein receptor-1
LPS	Lipopolysaccharides
M1	Macrophage type 1
M2	Macrophage type 2
MAPK	Mitogen-activated protein kinase
MCP-1	Monocyte chemoattractant protein 1
MCT1	Monocarboxylate transporter 1
MCT4	Monocarboxylate transporter 4
MD-2	Myeloid differentiation factor 2
MDA	Malondialdehyde
MRSA	Methicillin-resistant *Staphylococcus aureus*
mtROS	Mitochondrial reactive oxygen species
MUC2	Mucin 2
MyD88	Myeloid differentiation primary response 88
NADPH	Nicotinamide adenine dinucleotide phosphate
NEU1	Neuraminidase 1
NET	Neutrophil extracellular trap
NF-κB	Nuclear factor kappa B
NK	Natural killer
NKT	Natural killer T
NLRP3	NLR family pyrin domain containing 3
NO	Nitric oxide
NOD1	Nucleotide-binding oligomerization domain 1
NOD1/2	Nucleotide-binding oligomerization domain 1/2
NOD2	Nucleotide-binding oligomerization domain 2
NPC1L1	Niemann-Pick C1-like 1
NR4A1	Nuclear receptor subfamily 4 group A member 1
NR4A2	Nuclear receptor subfamily 4 group A member 2
Nrf2	Nuclear factor erythroid 2-related factor 2
Olfr78	Olfactory receptor 78
OSTα/OSTβ	Organic solute transporter alpha/beta
oxLDL	Oxidized low-density lipoprotein
*P. gingivalis*	*Porphyromonas gingivalis*
PAF	Platelet-activating factor
PAMPs	Pathogen-associated molecular patterns
PD-L1	Programmed death-ligand 1
PEPCK	Phosphoenolpyruvate carboxykina
Biomarker	A measurable biological parameter that reflects physiological or pathological processes, or response to a therapeutic intervention.
Dysbiosis	An imbalance in gut microbiota composition, diversity, or function, associated with disease states.
Eubiosis	A balanced microbial community structure compatible with health and homeostasis.
Fecal microbiota transplantation (FMT)	Therapeutic transfer of stool from a healthy donor to a patient in order to restore microbiota balance.
Gut microbiota	The community of microorganisms (bacteria, viruses, fungi, archaea) inhabiting the human gastrointestinal tract.
Microbiota-derived metabolites	Small molecules produced by microbial metabolism (e.g., TMAO, SCFAs, bile acids) that can affect host physiology.
SCFAs (Short-chain fatty acids)	Fermentation products of dietary fibers by gut microbiota, including acetate, propionate, and butyrate.
TMAO (Trimethylamine N-oxide)	A gut microbiota-derived metabolite from dietary choline and carnitine, associated with cardiovascular risk.
